# Supplementary material for: Multiple cAMP/PKA complexes at the STIM1 ER/PM junction specified by E-Syt1 and E-Syt2 reciprocally gates ANO1 (TMEM16A) via Ca2+
Source: Nat Commun. 2025 Apr 9;16:3378. doi: 10.1038/s41467-025-58682-w (PMC11982563; doi:10.1038/s41467-025-58682-w)

Multiple cAMP/PKA Complexes at the STIM1 ER/PM Junction, Specified by E-Syt1 and E-Syt2, Reciprocally Gates ANO1 (TMEM16A) via Ca^{2+}

Wei-Yin Lin[1], Woo Young Chung[1], Seonghee Park[2], Ava Movahed Abtahi[1], Benjamin Leblanc[1], Malini Ahuja[1] and Shmuel Muallem,[1, 3]

From [1]The Epithelial Signaling and Transport Section and The National Institute of Dental and Craniofacial Research, National Institutes of Health, Bethesda, MD 20892 and [2]Department of Physiology, Ewha Womans University College of Medicine, Seoul 07084, Korea.

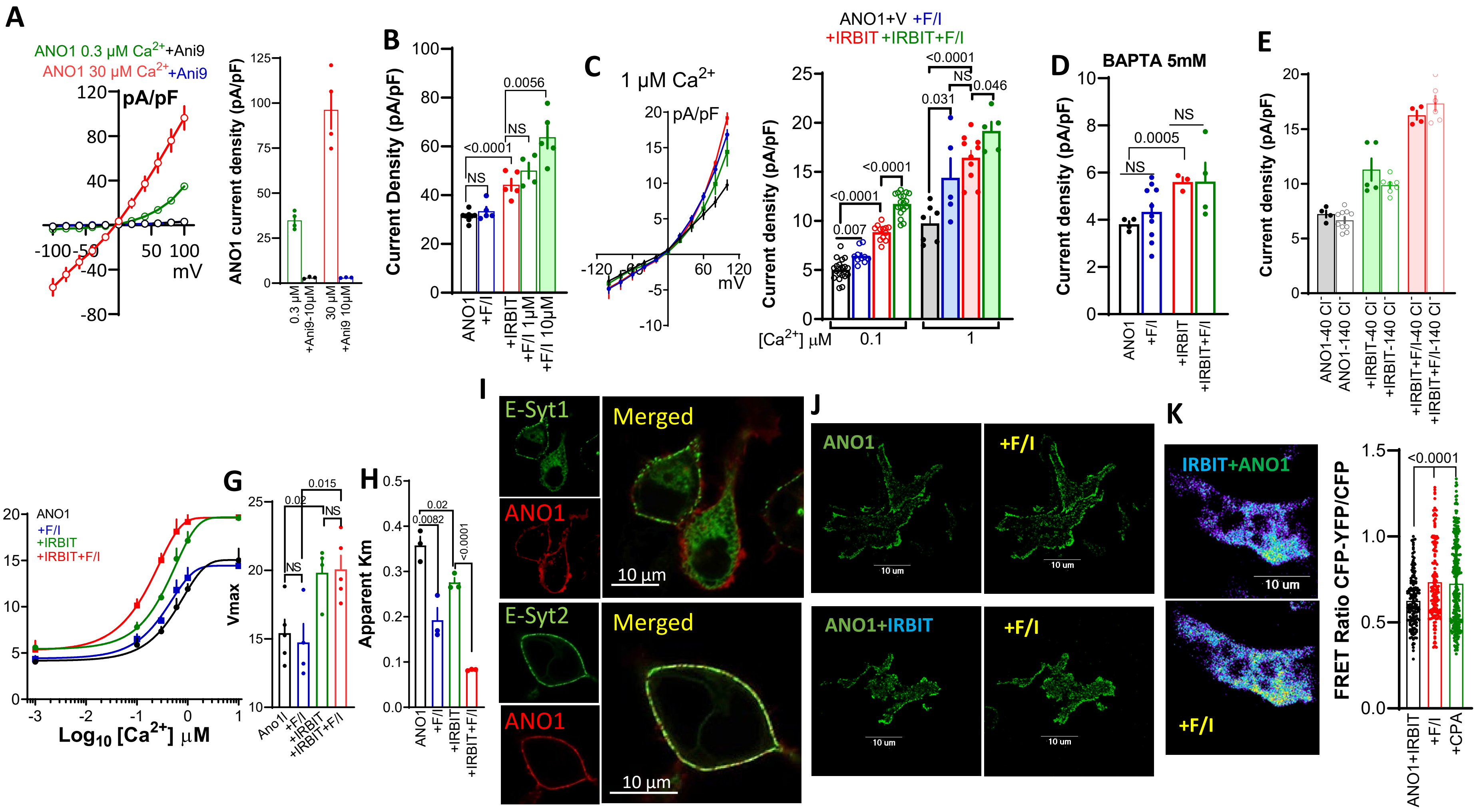

Supplementary Fig. 1: (A-B), Current was measured in HEK cells transfected with high level of ANO1 in the presence and absence of 10 μM Ani9 (A) and in the presence of IRBIT and stimulated with 1 or 10 μM F/I (B). **(C-D)**, HEK cells transfected with low level of ANO1 and empty vector (black) or IRBIT (red) were treated with vehicle (black, red) or 1 μM forskolin and 10 μM IBMX (F/I, blue, green). Current was measured with pipette solution in which Ca^{2+} was buffered with 5 mM EGTA to 0.1 of 1 μM Ca2+ as indicated (C) or with 5 mM BAPTA and no added Ca^{2+} (D "0 Ca^{2+}" condition). Shown are example I/Vs and average current density. **(E)**, Luck of effect of intracellular Cl^- concentration on ANO1 current. **(F-H)**, The total ANO1 currents of Figures 2B-D. **(I)**, Example confocal images of localization of ANO1-mCherry with E-Syt1-GFP and E-Syt2-GFP. **(J)**, Example of TIRF microscopy images for the analysis of surface ANO1 in Figure 2D. **(K)**, Example images and averages of FRET of ANO1-GFP and IRBIT-CFP measured before (black) and after stimulation with F/I (red) or treatment with 25 μM CPA for 7 min in Ca^{2+}-free media (green.

A

Position	Kinase	PSP	Score	Cutoff
74	AGC/PKA	VYHHKRASGSRTLAR	0.2156	0.1447
106	AGC/PKA	QPLPGKGSPVDAGSP	0.1485	0.1447
216	AGC/PKA	VAEHRPQTTKRLSYP	0.2069	0.1447
221	AGC/PKA	PQTTKRLSYPFSREK	0.1554	0.1447
466	AGC/PKA	EARVLEKSLRKESRN	0.1837	0.1447
471	AGC/PKA	EKSLRKESRNKETDK	0.2978	0.1447
537	AGC/PKA	VRSNIRVTVTATAVI	0.1517	0.1447
673	AGC/PKA	YLKLRRQSPSDREEY	0.3044	0.1447
675	AGC/PKA	KLRRQSPSDREEYVK	0.2082	0.1447
894	AGC/PKA	PDIPKDISQQIHKEK	0.148	0.1447

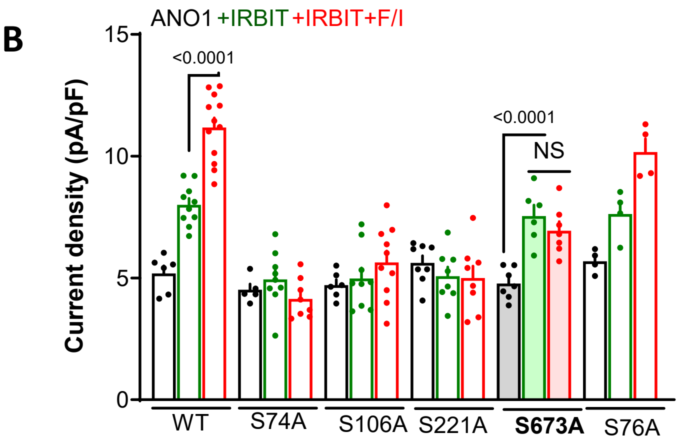

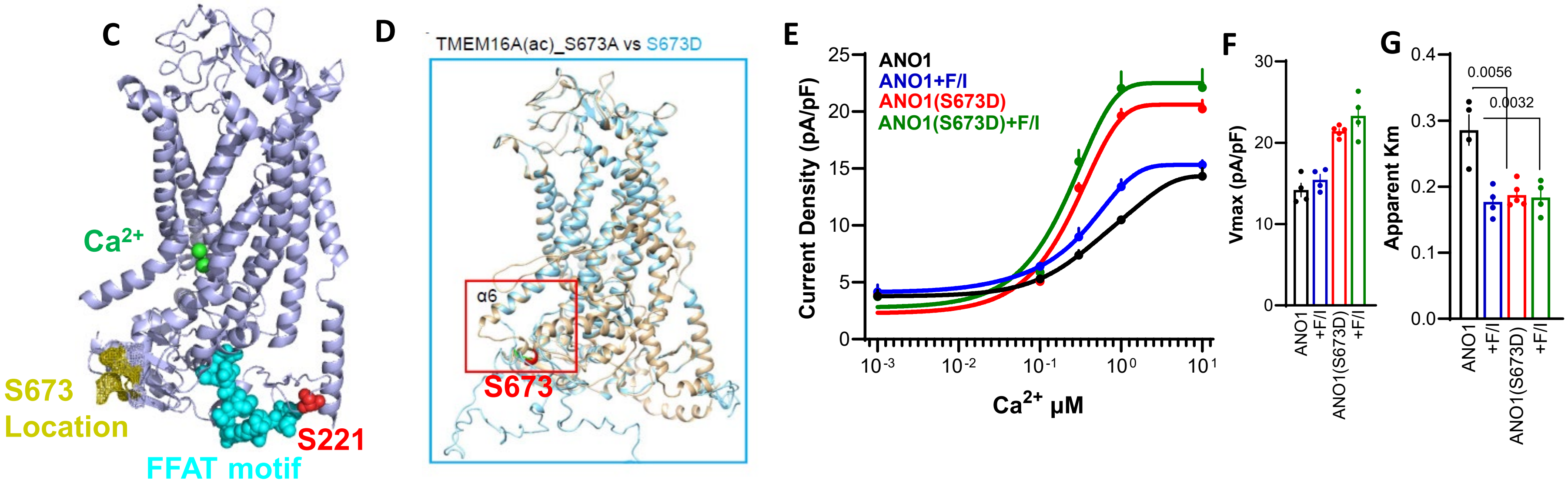

Supplementary Figure 2: (A), The PKA phosphorylation sites predicted by Group-based Prediction System (GPS) 6.0. **(B)**, Current was measured with the indicated ANO1 mutants expressed with vector (black) and IRBIT (green) and stimulated with F/I (red). **(C)**, ANO1 structure (PDB: 5OYB) with 2 Ca^{2+} ions (green), the predicted location of S673 (dark yellow mesh), the FFAT motif (turquoise) and S221 (red). **(D)**, the structure of TMEM16A (ac) predicted in Ko et al., PMID: 33199590 **(E-G)**, The total ANO1 currents of Figures 2J-L.

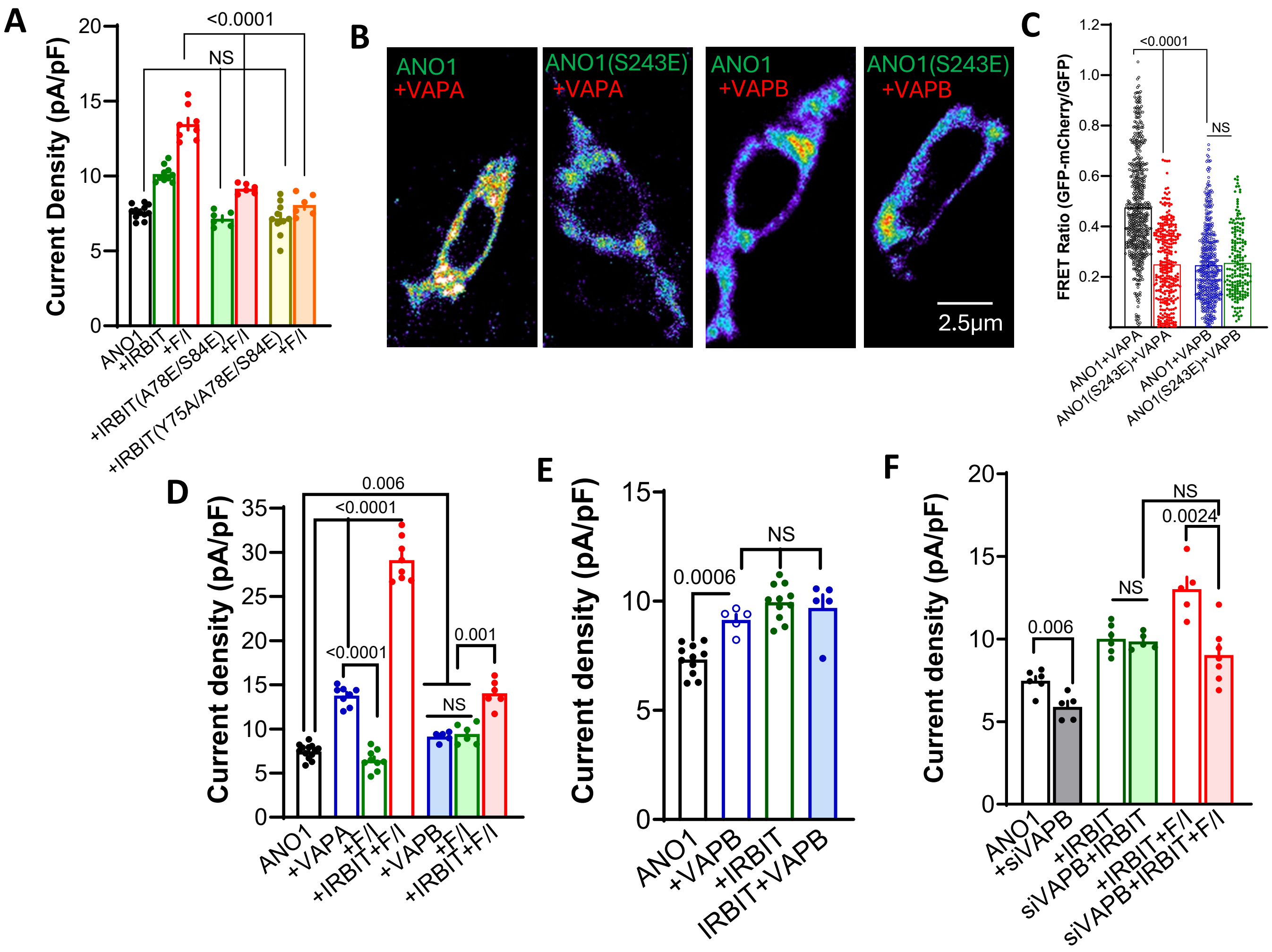

Supplementary Figure 3: (A), ANO1 current was measured in the presence of IRBIT (green, open column) IRBIT(A78E/S84E) (green, filled column), or IRBIT(Y75/A/A78E/S84E) (dark yellow), and stimulated with F/I (red, orange). **(B-C)** Example images and averages of FRET ratios between ANO1 or the ANO1 VAP mutant ANO1(S243A) and VAPA or VAPB. **(D)**, ANO1 current was measured in the presence of VAPA (open columns) or VAPB (filled columns), alone (blue) or with IRBIT (red) and stimulated with F/I (green, red). **(E)**, ANO1 current was measured with VAPB alone (blue, open column) , IRBIT alone (green) or VAPB+IRBIT (blue, filled column). **(F)**, ANO1 current was measured in cells treated with scrambled siRNA (open columns) or siVAPB and expressing ANO1 and vector (black) and IRBIT (green) and stimulated with F/I (red). Currents were measured at 0.3 μM Ca^{2+}.

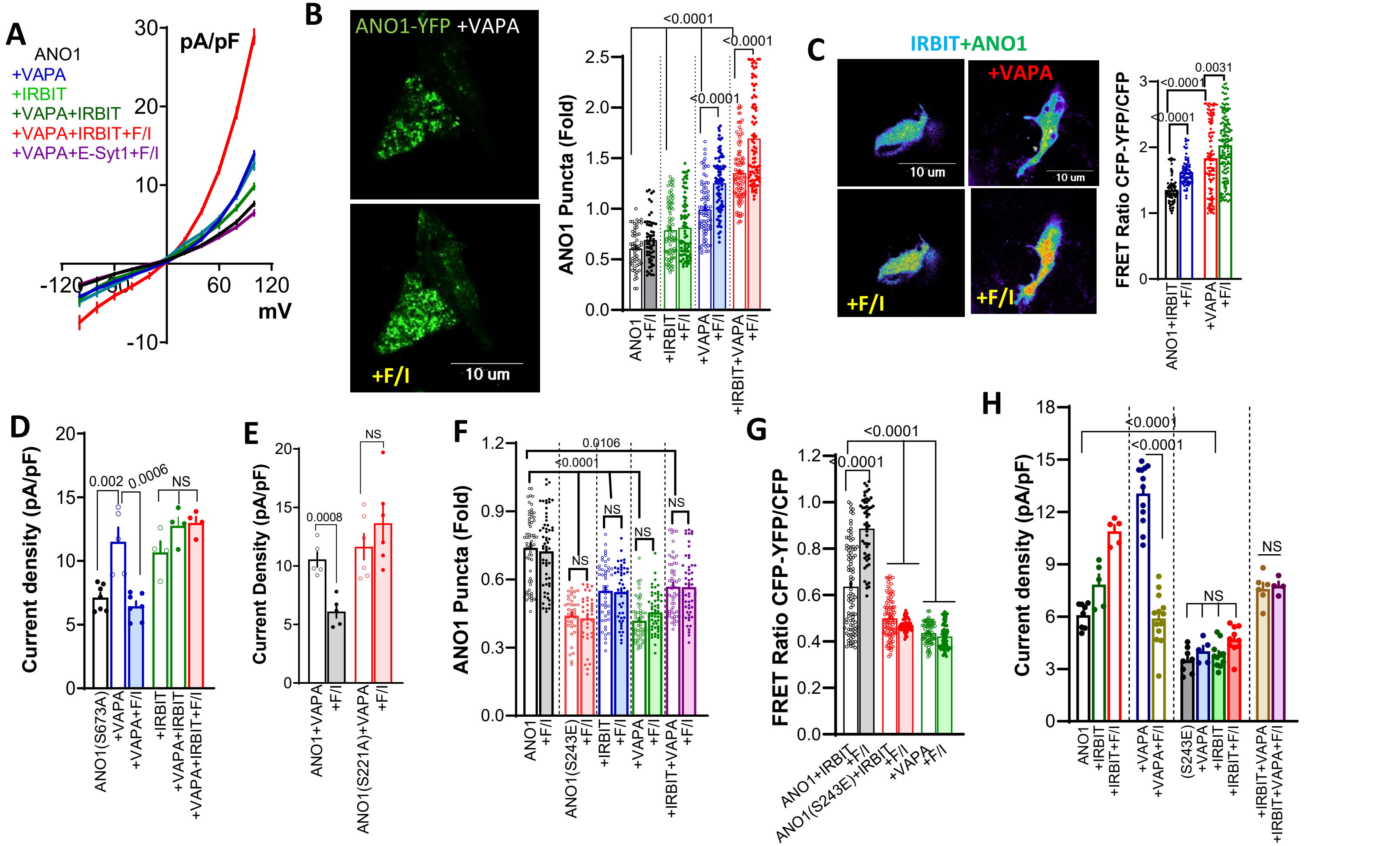

Supplementary Figure 4: (A) Example I/Vs for Figure 3A. **(B)** Example images and averages of the effect of IRBIT (green) VAPA (blue) and VAPA+IRBIT (red) before (open columns) and after stimulation with F/I (filled columns) related to Figure 3D-F. **(C)**, ANO1-YFP and IRBIT-CFP FRET (black) was measured in the presence of VAPA (red) and stimulated with F/I (blue, green). **(D)**, Effect of VAPA (blue), IRBIT (green, open symbols) IRBIT+VAPA (green, close symbols) and stimulated with F/I, were measured in cells expressing ANO1(S673A). **(E)**, Effect of VAPA was measured in cells expressing ANO1 (black) or ANO1(S221A) and stimulated with F/I (filled columns). **(F)**, Surface expression of ANO1(S243E) was measured in cells transfected with vector (red), IRBIT (blue) VAPA (green) and VAPA+IRBIT (purple) and treated with F/I (filled columns) .**(G)**, FRET was measured between ANO1-YFP and IRBIT-CFP (black) and ANO1(S243E)-YFP and IRBIT-CFP (red) and with VAPA (green) and stimulated with F/I (filled columns). **(H)**, Current was measured with ANO1 (open columns) or ANO1(S243E) (close columns) in the presence of IRBIT (green) VAPA (blue and dark yellow), IRBIT+VAPA (brown) and stimulated with F/I (red, dark yellow and purple).

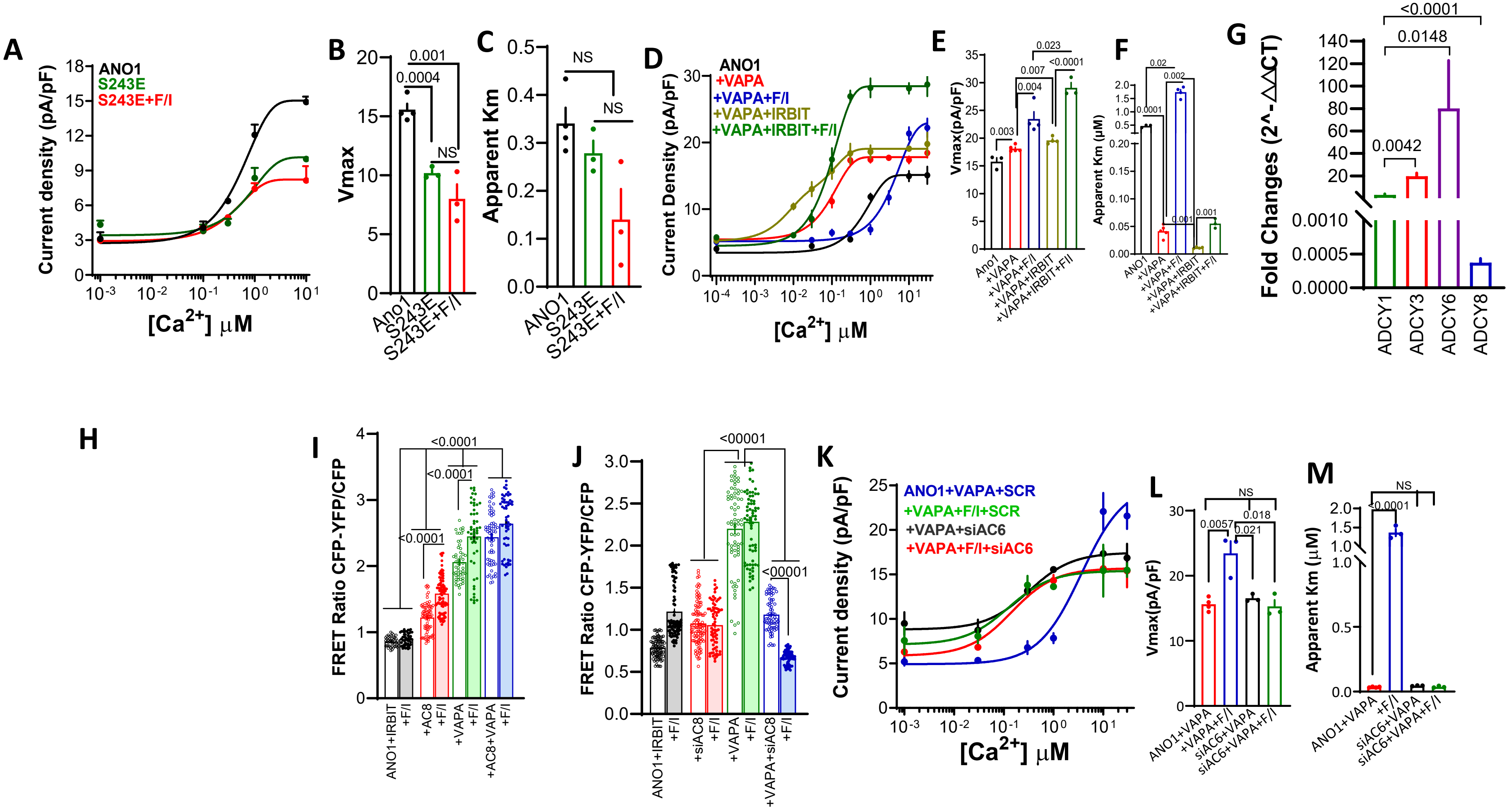

Supplementary Figure 5: (A-C), The total ANO1 currents of Figures 3G-I. **(D-F)**, The total ANO1 currents of Figures 3J-L. **(G-H)**, The mRNA level of AC1, AC3, AC6 and AC8 in HEKT cells (H) and their reduction by siRNA (I) were determined by QRT-PCR. **(I)**, ANO1-YFP and IRBIT-CFP FRET (black) was measured in the presence of AC8 (red), VAPA (green) and AC8+VAPA and stimulated with F/I (filled columns). **(J)**, ANO1-YFP and IRBIT-CFP FRET (black) was measured in cells treated with scrambled siRNA (black and green) or siAC8 (red, blue) and expressing vector (black, red) or VAPA (green, blue) and stimulated with F/I (filled columns). **(K-M)**, The total ANO1 currents of Figures 3N-P.

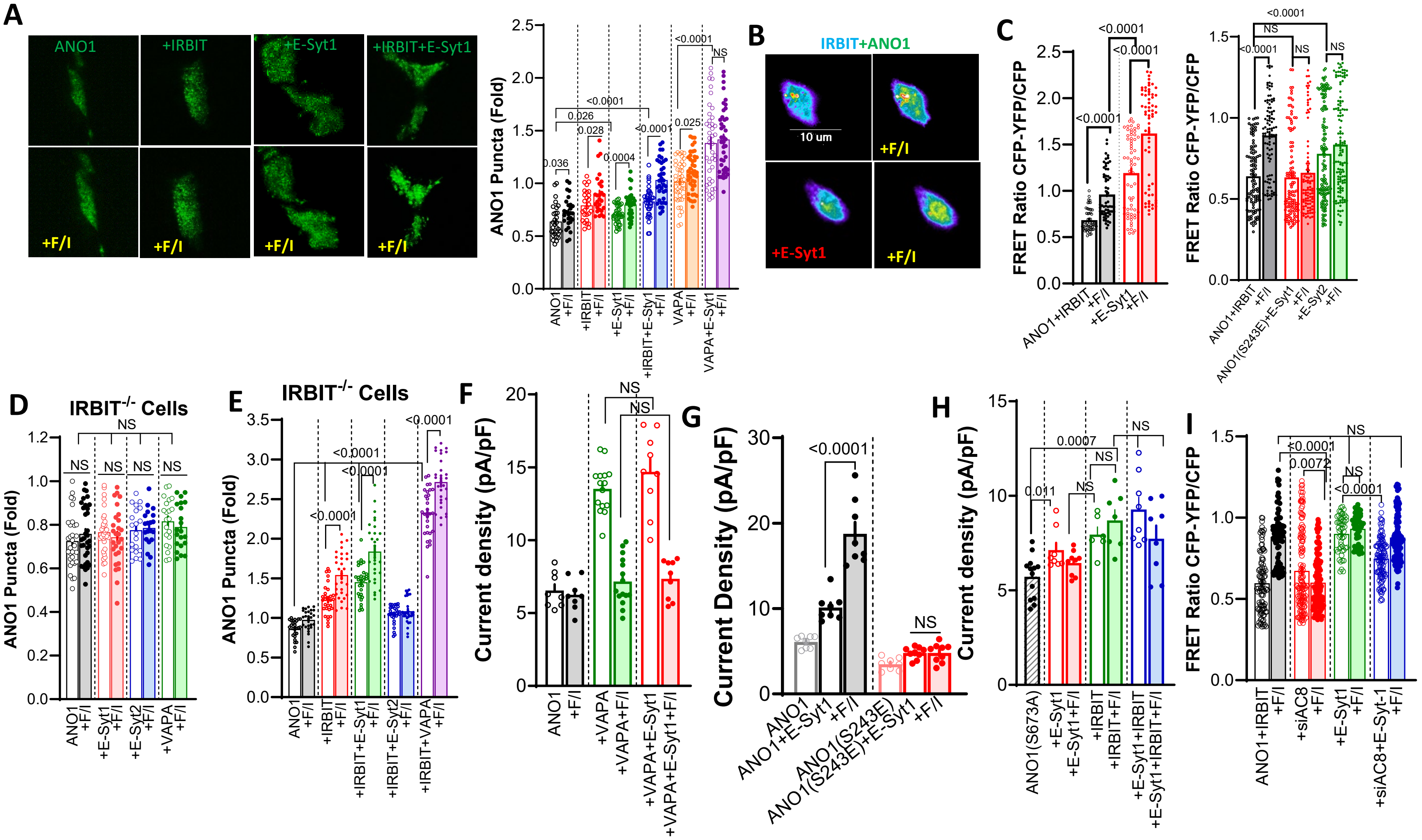

Supplementary Figure 6: (A), Example images and averages of ANO1 surface expression (black) in the presence of IRBIT (red), E-Syt1 (green), IRBIT+E-Syt1 (blue), VAPA (orange) or VAPA+E-Syt1 (purple) and stimulated with F/I (filled columns). **(B-C)**, Example images (B) and average FRET between ANO1-YFP or ANO1(S243E)-YFP and IRBIT-CFP (black) and in the presence of E-Syt1 (red), or E-Syt2 green) before (open columns) and after F/I treatment (filled columns. **(D)**, ANO1 in the TIRF field measured in IRBIT$^{-/-}$ cells related to Fig. 4C. **(E)**, ANO1 in the TIRF field was measured in IRBIT$^{-/-}$ cells, related to Figure 4B and 4E. **(F)**, Current was measured in cells expressing ANO1 (black) and VAPA (green) or VAPA+E-Syt1 (red) and treated with F/I (filled columns). **(G)**, current was measured in cells expressing ANO1 (black) or ANO1(S243E) (red) and E-Syt1 and treated with F/I (filled columns), as indicated. The current with ANO1 and ANO1(S243E) alone includes currents from Figure 3. **(H)**, Current was measured with ANO1(S673A) (black) and E-Syt1 (red), IRBIT (green) and IRBIT+E-Syt1 (blue) treated with F/I (filled columns). **(I)**, FRET was measured with ANO1-YFP and IRBIT-CFP in cells treated with scrambled siRNA (black), siAC8 (red, blue) expressing E-Syt1 (green, blue), and treated with F/I (filled columns).

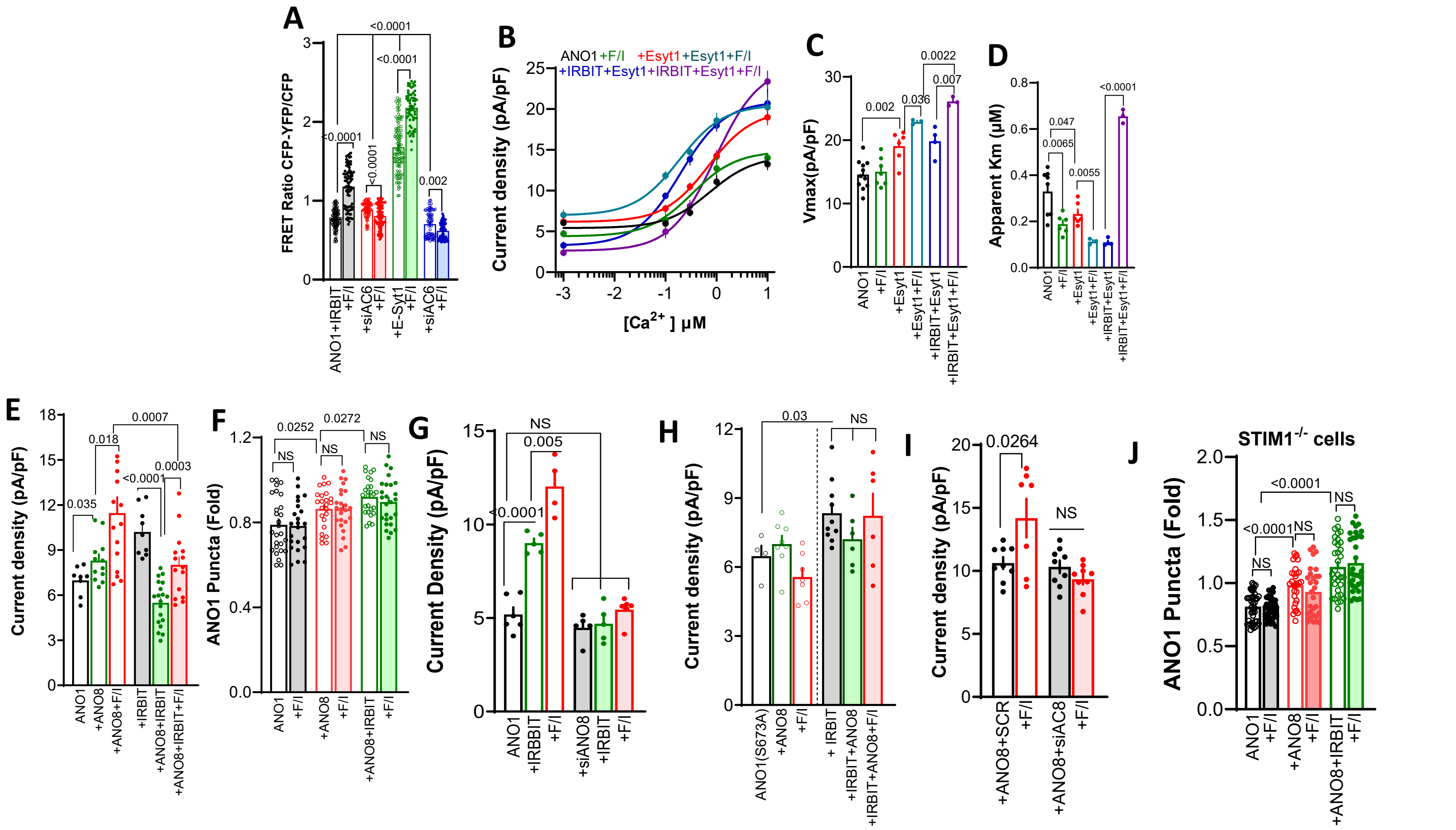

Supplementary Figure 7: (A), ANO1-YFP and IRBIT-CFP FRET (black) was measured in cells treated with scrambled siRNA (black and green) or siAC6 (red, blue) and expressing vector (black, red) or E-Syt1 (green, blue) and stimulated with F/I (filled columns). **(B-D),** The total ANO1 currents of Figures 4H-J. **(E)**, Current was measured without (open columns) or with transfected IRBIT (filled columns) in cells expressing anoctamin 8 (ANO8) (green) and stimulated with F/I (red). **(F)**, Surface ANO1 was measured in cells transfected with vector (black), ANO8 (red) or ANO8+IRBIT (green) and treated with F/I (filled columns). **(G)**, Cells treated with scrambled siRNA (open columns) or siANO8 (filled columns) were used to measure ANO1 current (black) and IRBIT (green) and treated with F/I (red). **(H)**, Current was measured without (open columns) or with transfected IRBIT (filled columns) in cells expressing ANO1(S695A) and ANO8 (green) and stimulated with F/I (red). (**I)**, Cells treated with scrambled siRNA (open columns) or siAC8 (filled columns) and expressing ANO8 were used to measure ANO1 current (black) and treated with F/I (red). **(J)**, Surface ANO1 was measured in STIM1$^{-/-}$ cells (black) expressing ANO8 (red) or ANO8+IRBIT (green) and treated with F/I (filled columns). Currents were measured at 0.3 μM Ca^{2+}.

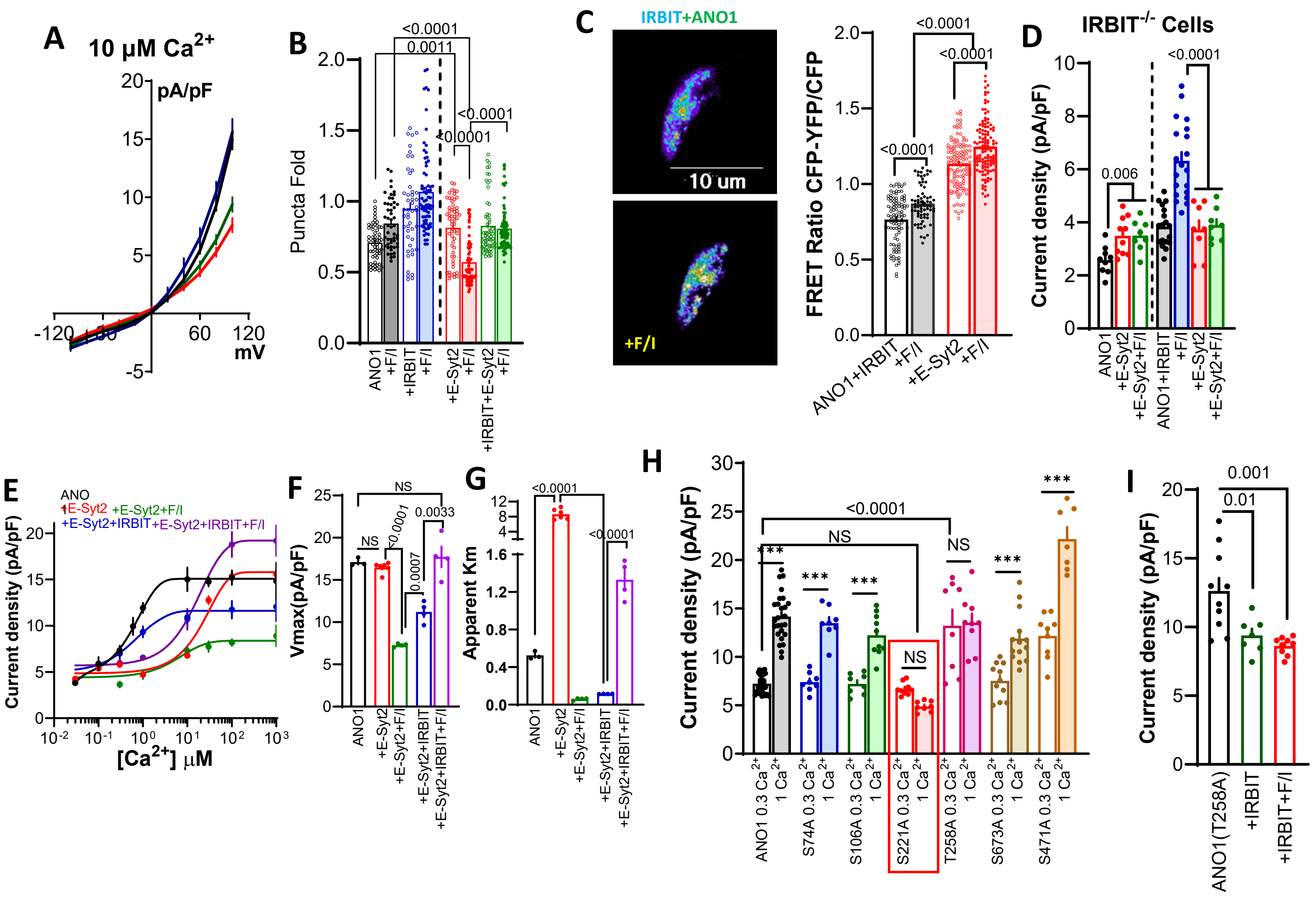

Supplementary Figure 8: (A), I/V plots for the current in Figure 5A. **(B),** Effect of F/I (filled columns), IRBIT (Blue) and E-Syt2 (red, green) on ANO1 puncta. **(C),** The ANO1-YFP and IRBIT-CFP FRET (black) was measured in cells expressing E-Syt2 (red) and treated with F/I (filled columns). **(D)**, Current was measured in IRBIT$^{-/-}$ cells (open columns) re-transfected with IRBIT (filled columns) expressing ANO1 (black) and E-Syt2 (red) and stimulated with F/I (green, blue). **(E-G)**, The total ANO1 currents of Figures 5D-F. **(H)**, Current was measured at 0.3 or 1 μM Ca^{2+} with indicated ANO1 predicted PKA phosphorylation site mutants. **(I)**, Current was measured at 0.3 μM Ca^{2+} with ANO1(T258A) (black) with IRBIT (green) and stimulated with F/I (red).

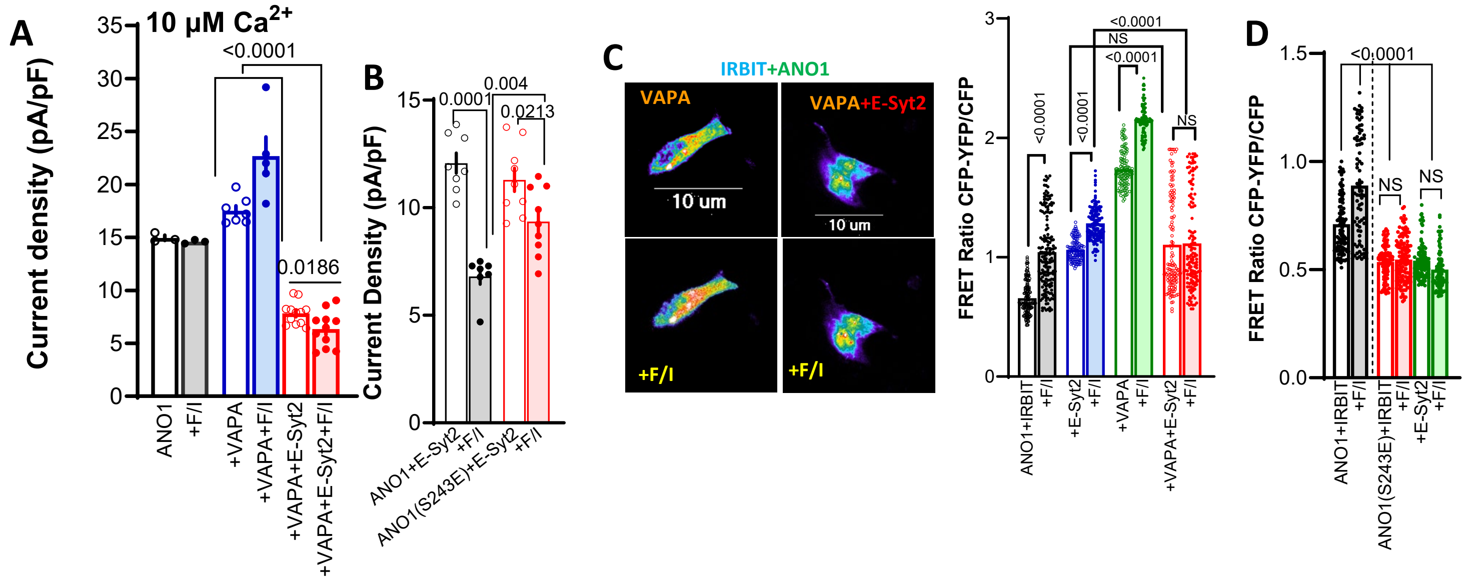

Supplementary Figure 9: (A), Current was measured with pipette solution buffered to 10 µM Ca^{2+} in cells expressing ANO1 (black), VAPA (blue) and VAPA+E-Syt2 (red) and treated with F/I (filled columns). (**B**), ANO1 (black) and ANO1(S243E) (red) currents were measured in the presence of E-Syt2 and treated with F/I (filled columns). (**C**), Example images and averages of ANO1-YFP and IRBIT-CFP FRET was measured in cells expressing ANO1 (black) and E-Syt2 (blue), VAPA (green) and VAPA+E-Syt2 (red). (**D**), FRET was measured with ANO1(S243E)-YFP and IRBIT-CFP (red) in the presence of E-Syt2 (green) with cells treated with F/I (filled columns)

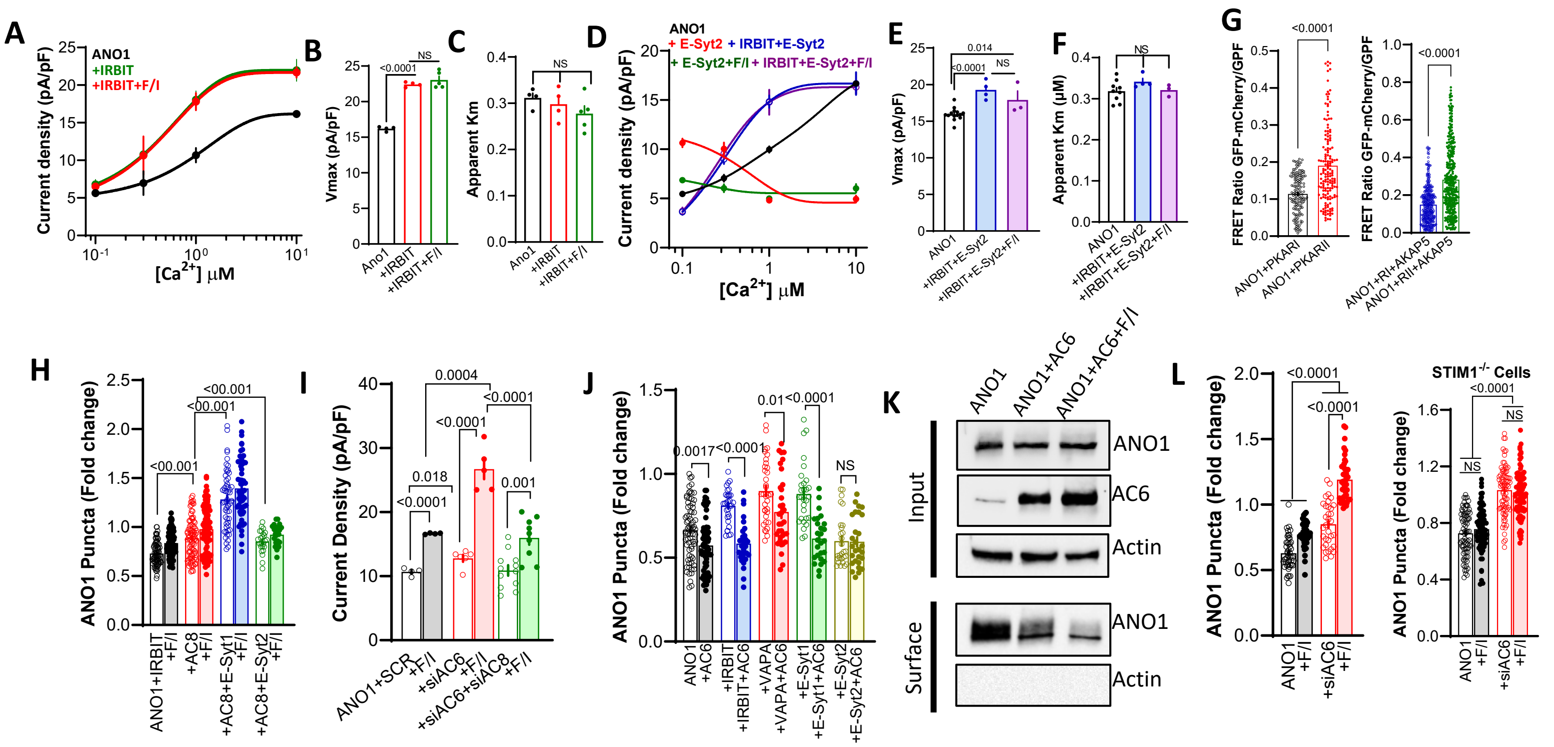

Supplementary Figure 10: (A-C), The total ANO1 currents of Figures 6G-I. **(D-F)**, The total ANO1 currents of Figures 6L-N. **(G)**, FRET between ANO1-mCherry and the GFP-tagged PKA regulatory subunits RI and RII. **(H)**, Effect of AC8 on ANO1 puncta in the presence and absence of E-Syt1 and E-Syt2. **(I)**, Current was measured in cells treated with scrambled siRNA (black), siAC6 (red) or siAC6+siAC8 (green) and stimulated with F/I (filled columns). **(J)**, Effect of AC6 on ANO1 level at the TIRF field in cells expressing vector, IRBIT, VAPA, E-Syt1 and E-Syt2. **(K)**, Effect of AC6 on surface ANO1. **(L)** Depletion of AC6 increases ANO1 level at the TIRF field. **(L),** ANO1 at the TIRF field was measured in STIM1$^{-/-}$ cells treated with siAC6.

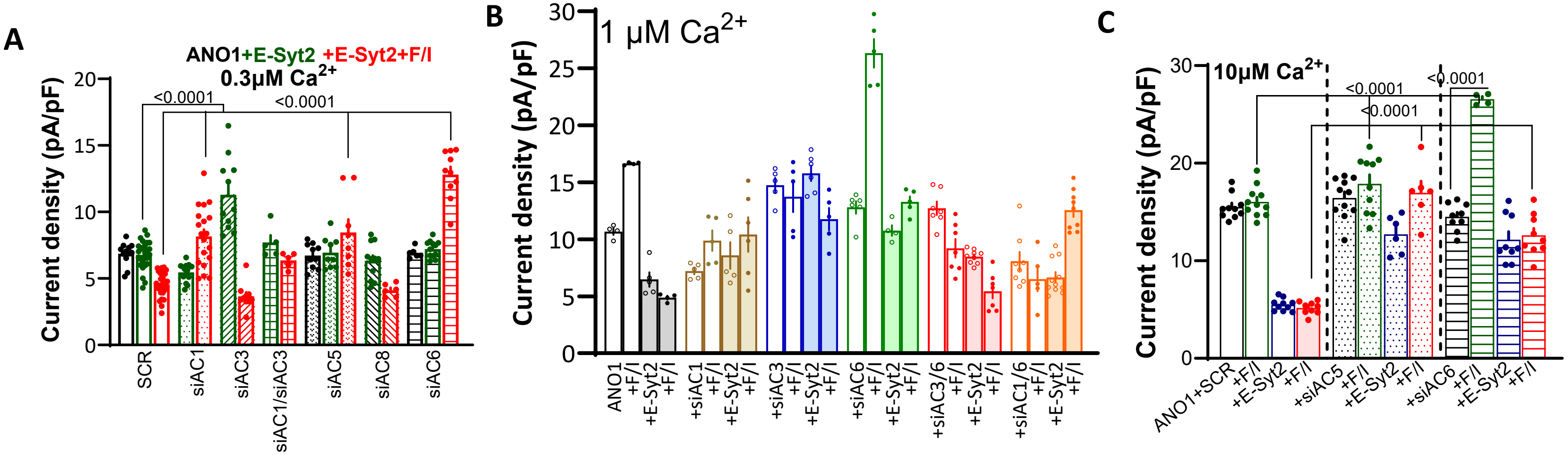

Supplementary Figure 11: (A), Current was measured in cells treated with scrambled siRNA (open column, siAC1, siAC3, siAC1+siAC3, siAC5, siAC8 or siAC6, as indicated, and expressing ANO1 (black) and E-Syt2 (green) and stimulated with F/I (red). **(B)**, The total ANO1 currents of Figures 7D. **(C)**, Current was measured at 10 μM Ca^{2+} in cells treated with scrambled siRNA(open columns), siAC5 (dotted columns) or siAC6 (striped columns) expressing E-Syt2 (blue) and stimulated with F/I (green, red).

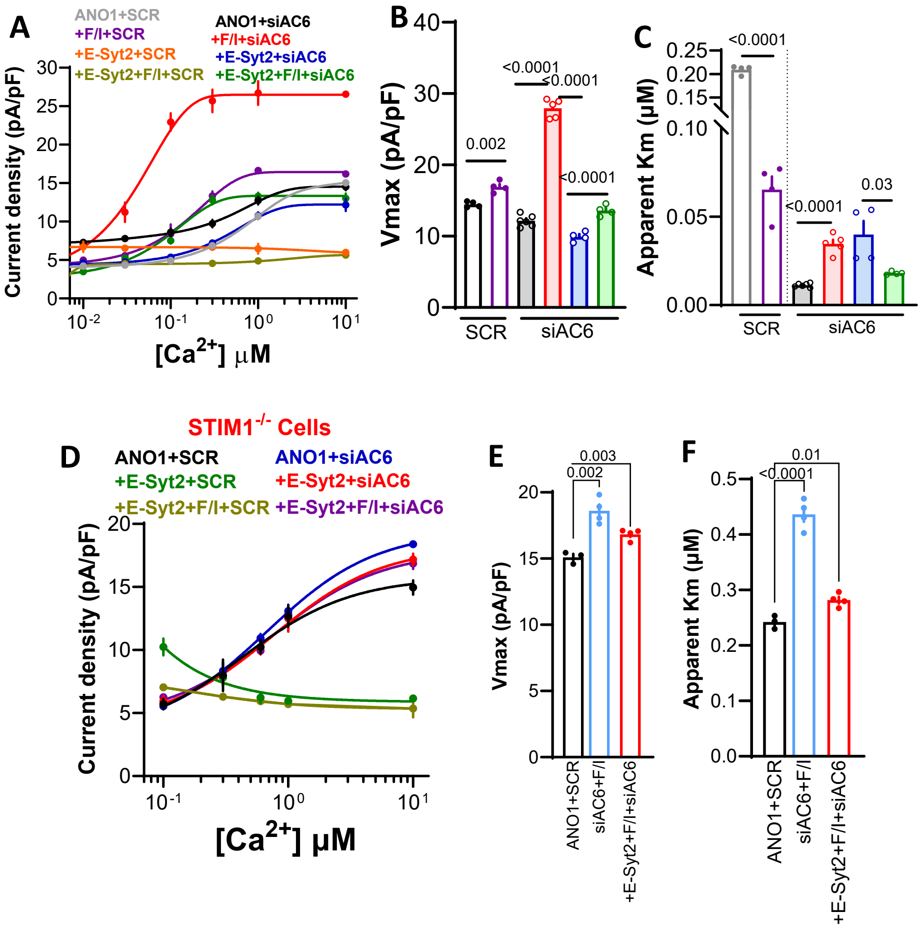

Supplementary Figure 12: (A-C), The total ANO1 currents of Figures 7E-G. **(D-F)**, The total ANO1 currents of Figures 7I-K.

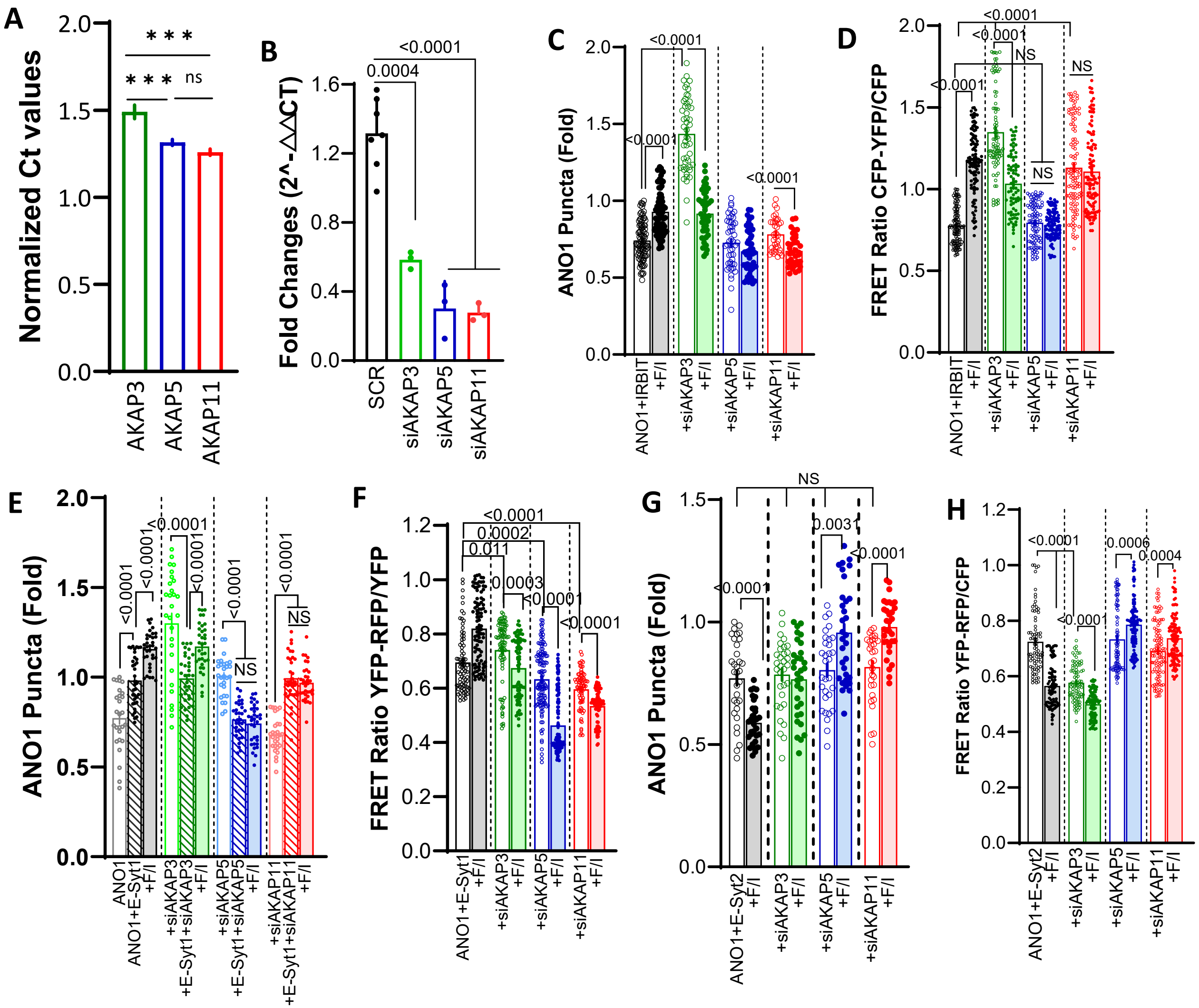

Supplementary Figure 13: (A-B), AKAP3,AKAP5 and AKAP11 mRNA (A) and effect of the siRNA were measured by QRT-PCR (B). **(C, E, G)**, Effect of depletion of the AKAPs on ANO1 at the TIRF field in the presence of IRBIT (C), E-Syt1 (E) or E-Syt2 (G) and stimulated with F/I (filled columns). **(D, F, H)**, Effect of depletion of the AKAPs on FRET between ANO1-irbir (D), ANO1 and E-Syt1 (F) or ANO1 and E-Syt2 (H) and stimulated with F/I (filled columns).

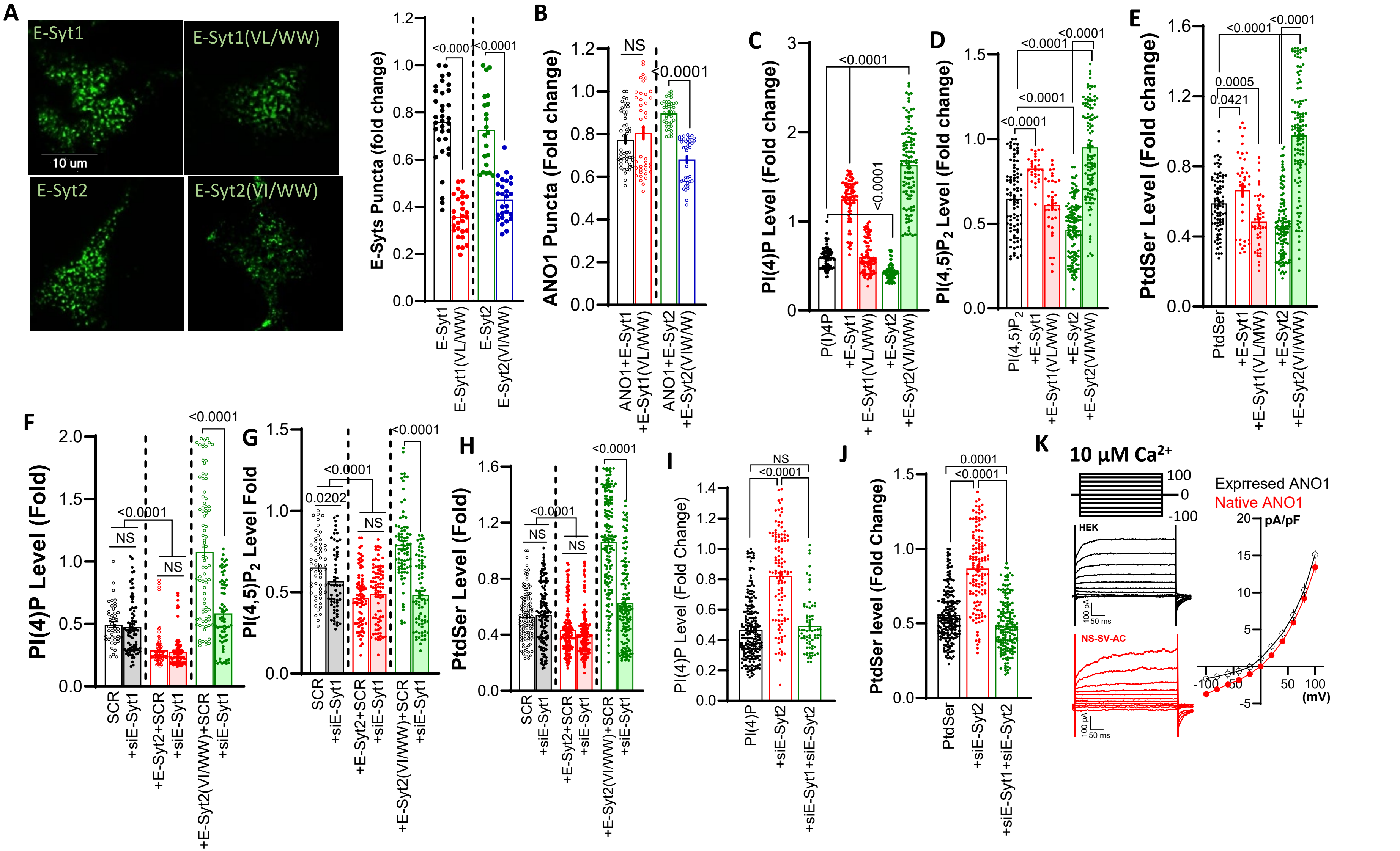

Supplementary Figure 14: (A), Near surface of GFP-tagged E-Syt1, E-Syt2 and the lipid transfer mutants E-Syt1(V169W/L308W) and E-Syt2(V197W/I337W) was evaluated by TIRF microscopy. Shown are example images and averages. **(B)**, Surface ANO1 was measured in cells expressing ANO1 and the indicated E-Syts. **(C-E)** Cells expressing E-Syt1, E-Syt2 and the lipid transfer mutants E-Syt1(V169W/L308W) and E-Syt2(V197W/I337W) and the specific lipid sensors (see methods) were used to measure PM PI(4)P (C), PI(4,5)P$_2$ (D) and PtdSer (E). **(F-H)** Cells treated with SCR or siE-Syt1 and transfected with vector (black) E-Syt2 (red) or E-Syt2(V197W/I337W) (green) were used to measure PM PI(4)P (F), PI(4,5)P$_2$ (G) and PtdSer (H). **(I-J)** Cells treated with SCR or siE-Syt2 (red) or siE-Syt2+siE-Syt1 (green) were used to measure PM PI(4)P (I) and PtdSer (J). **(K)** HEKT cells transfected with ANO1 and NS_SV-AV cells were used to measure ANO1 current at 10 μM Ca^{2+}.

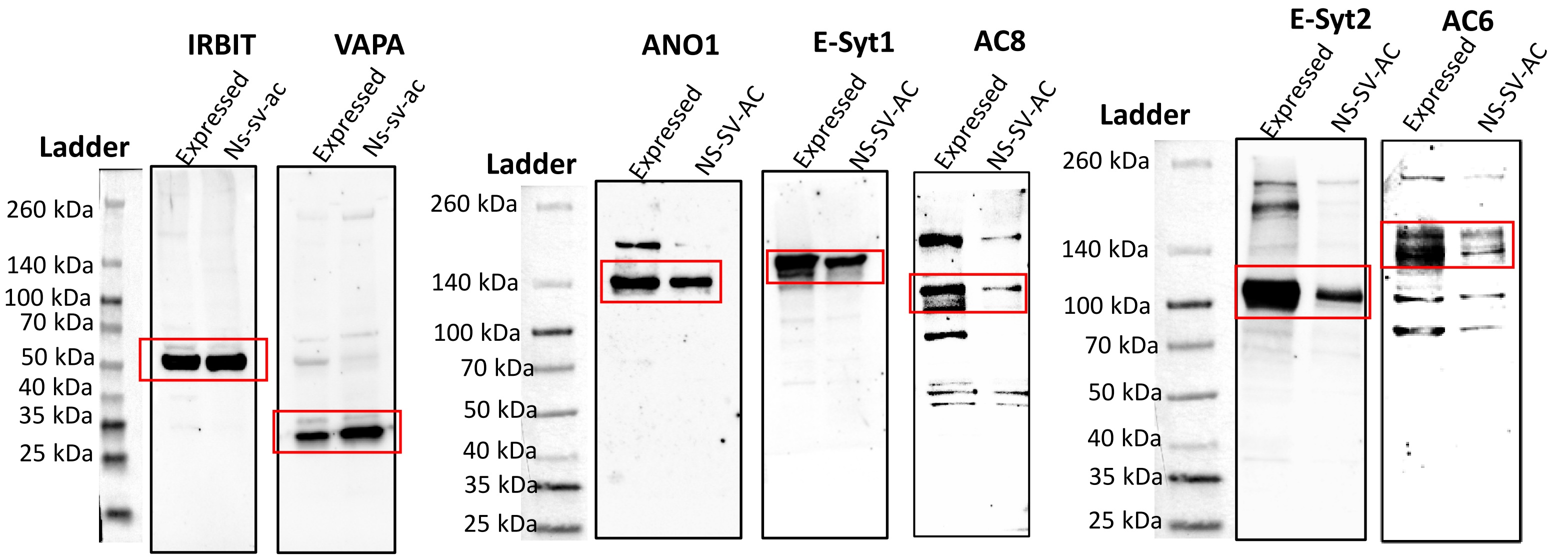

Supplementary Figure 15: Validation of the indicated antibodies and their use to determine expression of the proteins in the immortalized parotid acinar cell line NS-VS-AC

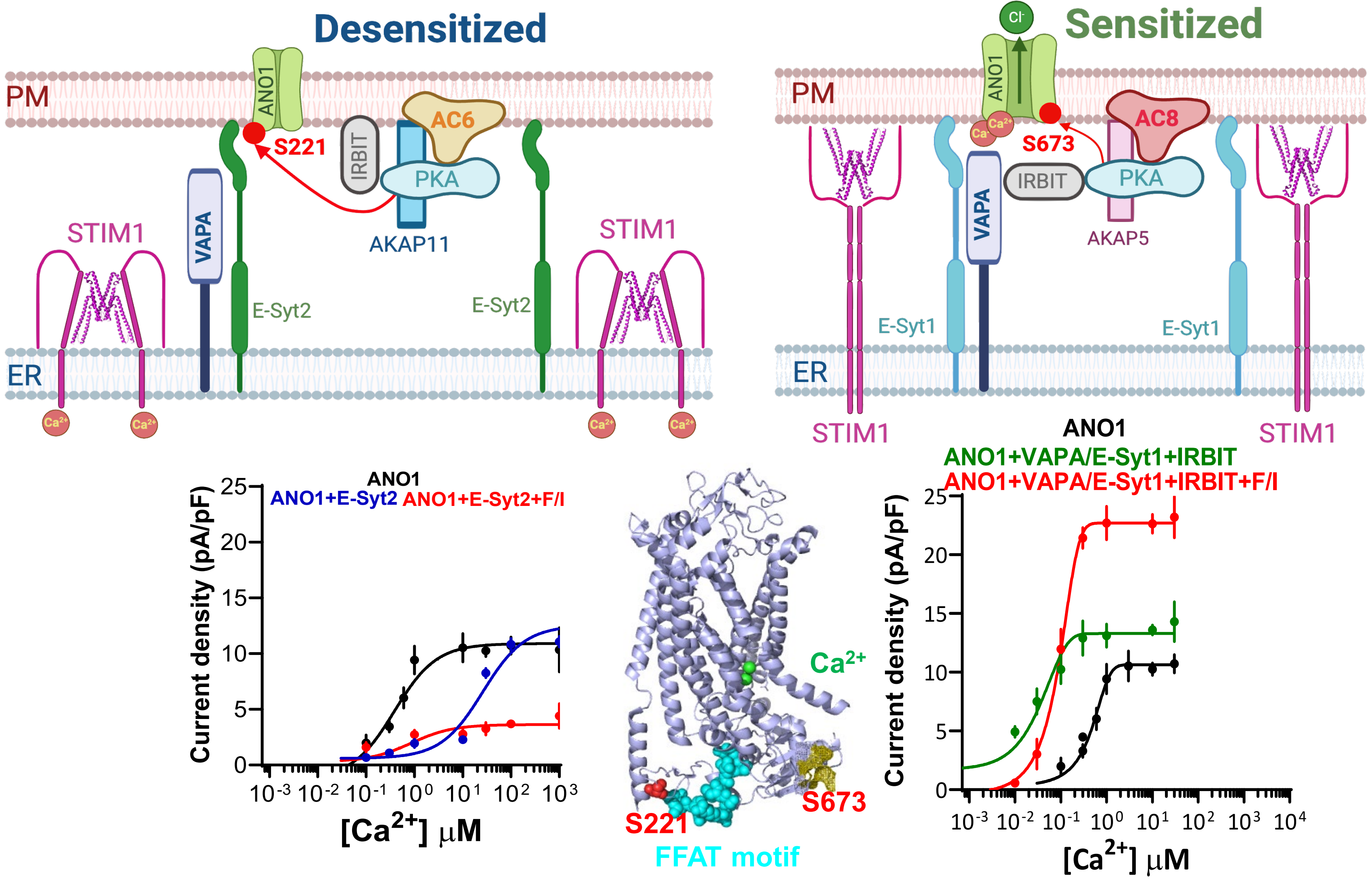

Supplementary Figure 16: At the resting, none-secretory state, E-Syt2 reduces junctional PI(4)P, PI(4,5)P_2 and PtdSer to preclude interaction of ANO1 with VAPA and localization of ANO1 at the PI(4,5)P_2-rich STIM1 junctions. E-Syt2 then recruits the AC6-AKAP11-PKA complex to phosphorylate ANO1 S221 and confer the ANO1 conformation with the low Ca^{2+} affinity state and ANO1 is desensitized.

Upon cell stimulation of Ca^{2+} mobilizing receptor and Ca^{2+} release from the ER, STIM1 unfolds to form the junction in which E-Syt1 set the level of PtdSer and the PI(4)P and PI(4,5)P_2-rich domain ER/PM junctions. Stabilization of STIM1 junction by interaction of STIM1 polybasic domain with PI(4)P PI(4,5)P_2 promotes interaction of VAPA/IRBIT with ANO1 and its interaction with AC8-AKAP3/5-PKA to phosphorylate ANO1 S695 and confer the ANO1 conformation with the high Ca^{2+} affinity state. ANO1 is sensitized to increase the luminal membrane Cl^- conductance and initiate the process of epithelial fluid and electrolyte secretion.

Supplementary Table 1 | Primers utilized for constructing plasmids. F stands for forward, while R stands for reverse.

ANO1(S74A)	F	5'- GTA TAC CAT CAC AAG AGA GCC GCC GGG AGC AGG ACT CTG GCC AGG-3'
	R	5'- CCT GGC CAG AGT CCT GCT CCC GGC GGC TCT CTT GTG ATG GTA TAC-3'
ANO1(S74D)	F	5'-TAC CAT CAC AAG AGA GCC GAC GGG AGC AGG ACT CTG GCC-3'
	R	5'- GGC CAG AGT CCT GCT CCC GTC GGC TCT CTT GTG ATG GTA-3'
ANO1(S76A)	F	5'- CAT CAC AAG AGA GCC TCA GGG GCC AGG ACT CTG GCC AGG AGG GGA-3'
	R	5'- TCC CCT CCT GGC CAG AGT CCT GGC CCC TGA GGC TCT CTT GTG ATG-3'
ANO1(S106A)	F	5'- CCC CTT CCC GGG AAG GGG GCC CCT GTG GAT GCA GGC TCA-3'
	R	5'- TGA GCC TGC ATC CAC AGG GGC CCC CTT CCC GGG AAG GGG-3'
ANO1(S221A)	F	5'- CCA CAG ACC ACA AAG AGG CTC GCC TAT CCC TTC TCC CGG GAG AAG -3'
	R	5'- CTTCTCCCGGGAGAAGGGATAGGCGAGCCTCTTTGTGGTCTGTGG -3'
ANO1 (S221D)	F	5’- CAG ACC ACA AAG AGG CTC GAC TAT CCC TTC TCC CGG GAG-3'
	R	5’-CTC CCG GGA GAA GGG ATA GTC GAG CCT CTT TGT GGT CTG-3'
ANO1(S243E)	F	5'-GAC TCT TTT TTC GAC GAA AAA ACC CGG AGC ACA-3'
	R	5'- TGT GCT CCG GGT TTT TTC GTC GAA AAA AGA GTC-3'
ANO1(T258A)	F	5'- ATC CTG AAG AGA ACA GCG TGC ACC AAG GCC AAG -3'
	R	5'- CTT GGC CTT GGT GCA CGC TGT TCT CTT CAG GAT- 3'
ANO1(S471A)	F	5'- TCA CTG AGA AAA GAA GCC AGA AAC AAA GAG ACC-3'
	R	5'- GGT CTC TTT GTT TCT GGC TTC TTT TCT CAG TGA-3'
ANO1(I636A)	F	5'- GGC TGC CTC ATG GAG CTC TGT GCC CAG CTG AGC ATC ATT ATG CTG-3'
	R	5'- CAG CAT AAT GAT GCT CAG CTG GGC ACA GAG CTC CAT GAG GCA GCC-3'
ANO1(Q646A)	F	5'- ATC ATT ATG CTG GGC AAG GCG CTA ATC CAG AAC AAT CTC-3'
	R	5'- GAG ATT GTT CTG GAT TAG CGC CTT GCC CAG CAT AAT GAT -3'
ANO1(S673A)	F	5'- TAC CTG AAG CTG CGC AGA CAG GCC CCC TCA GAC CGT GAA GAG TAC-3'
	R	5'- GTA CTC TTC ACG GTC TGA GGG GGC CTG TCT GCG CAG CTT CAG GTA-3'
ANO1(S673D)	F	5'- CTG AAG CTG CGC AGA CAG GAC CCC TCA GAC CGT GAA GAG-3'
	R	5'- CTC TTC ACG GTC TGA GGG GTC CTG TCT GCG CAG CTT CAG-3'
IRBIT(A78E/S84E)	F	5'- ACA GCA AGC TAC AGT TCA GAA GCA TCC TAT ACA GAT GAA TCT GAT GAT GAG GTT TCC -3'
	R	5'-GAG GGG AAA CCT CAT CAG ATT CAT CTG TAT AGG ATG CTT CTG AAC TGT AGC TGT CTG -3'
IRBIT(Y75A/A78E)	F	5'- TCA CAA TCC TCC ACA GAC AGC GCC AGT TCA GAA GCA TCC TAT ACA GAT AGC TCT GAT-3'
	R	5'-ATC AGA GCT ATC TGT ATA GGA TGC TTC TGA ACT GGC GCT GTC TGT GGA GGA TTG TGA-3'
E-syt1 V169W	F	F 5’-GCT GAA ACT GTG GCT CCG GCT TGG AGG GGA TCT AAC CCC CAT CTG
	R	5’-CAG ATG GGG GTT AGA TCC CCT CCA AGC CGG AGCC ACA GTT TCA GC

E-syt1 (L308W)	F	5’-TTC CTC GTG TTG CCC AAC CGA TGG CTG GTG CCC CTT GTG CCT GAC
	R	5’-GTCA GGC ACA AGG GGC ACC AGC CAT CGG TTG GGC AAC ACG AGG AA
E-syt2 (V197W)	F	5’-CGA GAA ACT ATA GAA CCA GCC TGGC GGG GAG CAA ACA CCC ACC TT
	R	5’-AAG GTG GGT GTT TGC TCC CCG CCA GGC TGG TTC TAT AGT TTC TCG
E-syt2 (I337W)	F	5’-TAT CTG GTG CTT CCC AAT CGA TGG ACC GTT CCA CTT GTC AGT GAA
	R	5’-TTC ACT GAC AAG TGG AAC GGT CCA TCG ATT GGG AAG CAC CAG ATA
E-Syt1 ΔC2E	F	5'-GAGGCTCCAGCCGGGGACCTGATGGACAACAAG
	R	5'-CTT GTT GTC CAT CAG GTC CCC GGC TGG AGC CTC-3'
E-syt2 (RRK/AAA)	F	5'-CCA GAC AAG AGG CGG TCA GGA GCG GCG GCA ACA CAC GTG TCA AAG AAA ACA TTA-3'
	R	5'-TAA TGT TTT CTT TGA CAC GTG TGT TGC CGC CGC TCC TGA CCG CCT CTT GTC TGG-3'

Supplementary Table 2 | Primers utilized for sequencing. F stands for forward, while R stands for reverse.

ANO1-1	F	5'- ATG AGG GTC CCC GAG AAG TAC TCG- 3'
ANO1-500	F	5'- TGC TCT GTA GGG AAG CTG AGT TTT TGA AAC- 3'
ANO1-1021	F	5'- CTG GTC AGG AAA TAC TTT GGT GAG AAG- 3'
ANO1-1800	F	5'-AGG CTA ACC TTC AAG GCC TTC CTGCTG CTC AAG- 3'
IRBIT	F	5'-GGA AAG AGG CGG GGG CGG CGG GTC AGC CGC - 3'
	R	5'- TTC TGA GTT GAG TAG ATG TTA CAA GCA GAC- 3'
E-Syt1 158	F	5’-CGG TGC TGA CTT CAT TCG-3'
E-Syt1	F	5’-CAA GAC TAT TTC GCA AAC-3'
E-Syt2 178	F	5’-TTC AGC TGG GTT CTC CTC-3'
E-Syt2	F	5’-TCC ACA CCA GTC ATT GGG-3'

Supplementary Table 3 | All siRNAs utilized in the current study.

Construct	Primers
hs.Ri.AC1	rGrGrArCrArUrGrArGrArGrGrArArCrArGrUrUrUrCrUrUGA
	rUrCrArArGrArArArCrUrGrUrUrCrCrUrCrUrCrArUrGrUrCrCrGrU
hs.Ri.AC3	rArUrGrCrUrCrArGrCrUrUrCrUrArCrUrArCrUrUrCrUrCCC
	rGrGrGrArGrArArGrUrArGrUrArGrArArGrCrUrGrArGrCrArUrCrA
hs.Ri.AC5	Dharmacon™ # L-006638-01-0005
hs.Ri.AC6	rGrGrUrCrArUrGrArCrCrCrUrGrArArUrGrArGrCrUrCrUTT
	rArArArGrArGrCrUrCrArUrUrCrArGrGrGrUrCrArUrGrArCrCrArG
hs.Ri.AC8	rGrGrCrGrArUrArArArUrUrGrArGrArArGrArGrArGrCrATA
	rUrArUrGrCrUrCrUrCrUrUrCrUrCrArArUrUrUrArUrCrGrCrCrArC
hs.Ri.E-Syt1	rCrUrCrUrArUrArUrGrArGrUrCrArUrCrGrArGrArGrCrUAC
	rGrUrArGrCrUrCrUrCrGrArUrGrArCrUrCrArUrArUrArGrArGrArG
hs.Ri.E-Syt2	rArGrUrCrArUrGrArGrCrUrUrUrGrUrUrArArUrUrUrUrCAA
	rUrUrGrArArArArUrUrArArCrArArArGrCrUrCrArUrGrArCrUrGrA
hs.Ri.VAPA	rCrArArUrUrGrUrGrArCrUrGrUrUrUrCrArGrUrArArUrGCT
	rArGrCrArUrUrArCrUrGrArArArCrArGrUrCrArCrArArUrUrGrArC
hs.Ri.VAPB	rGrArUrGrUrCrArGrUrUrGrArArUrArArArArCrArGrUrACT
	rArGrUrArCrUrGrUrUrUrUrArUrUrCrArArCrUrGrArCrArUrCrUrU
hs.Ri.ANO8 (Jha A. et al., 2019)	rGrUrGrGrArGrArGrCrGrArGrCrUrArCrGrCrUrUrCrUrUCA
	rUrGrArArGrArArGrCrGrUrArGrCrUrCrGrCrUrCrUrCrCrArCrArU
hs.Ri.AKAP3	rGrUrArCrCrCrUrGrArArGrArUrArUrUrGrGrCrArArCrCTC
	rGrArGrGrUrUrGrCrCrArArUrArUrCrUrUrCrArGrGrGrUrArCrUrC
hs.Ri.AKAP5	rCrUrCrUrArGrUrCrArArGrArArUrGrCrUrArUrUrCrArGTT
	rArArCrUrGrArArUrArGrCrArUrUrCrUrUrGrArCrUrArGrArGrArA
hs.Ri.AKAP11	rArGrCrArUrGrGrArArArCrCrUrUrGrArUrCrArArArArAAA
	rUrUrUrUrUrUrUrGrArUrCrArArGrGrUrUrUrCrCrArUrGrCrUrUrA
Scrabble	rCrGrUrArArUrCrGrCrGrUrArArUrArCrGrCrGrUrArT
	rArUrArCrGrCrGrUrArUrUrArCrGrCrGrArUrUrArArCrGrArC
hs.Ri.STIM1	rGrGrCrUrCrUrGrGrArUrArCrArGrUrGrCrUrCTT
	rGrArGrCrArCrUrGrUrArUrCrCrArGrArGrCrCTT
hs.Ri.IRBIT	rCrArArUrUrGrUrCrUrArCrArUrUrArCrCrGrArArArUrCGA
	rUrCrGrArUrUrUrCrGrGrUrArArUrGrUrArGrArCrArArUrUrGrCrU

Uncropped blots

Figure 2D

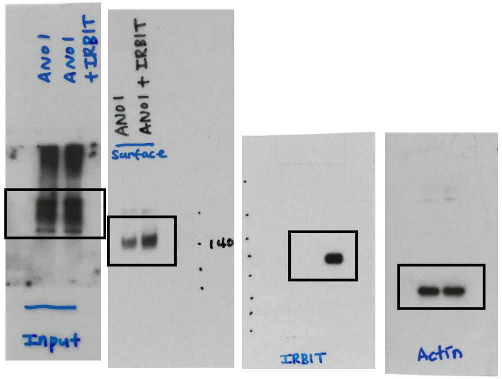

Figure 3D

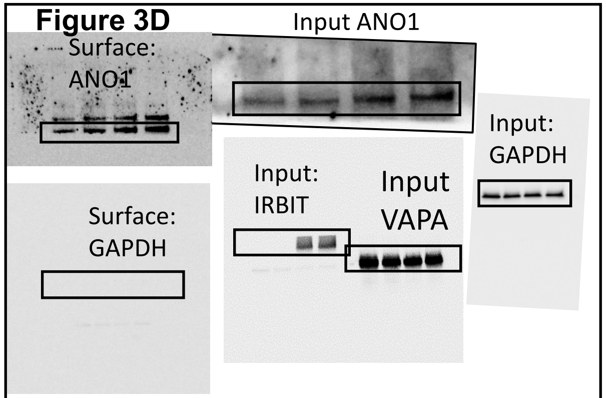

Figure 4D

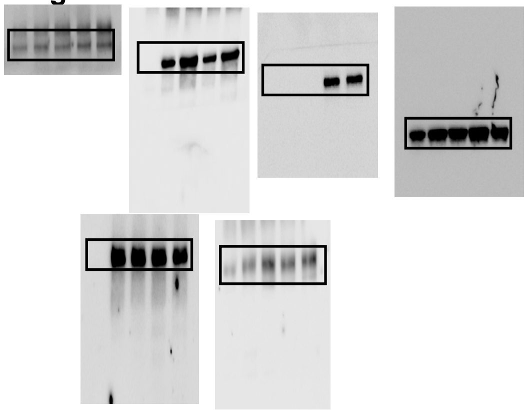

Figure 5B

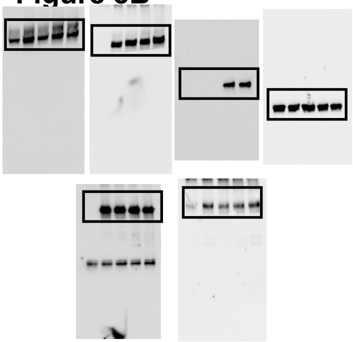

Supplementary Figure 10K

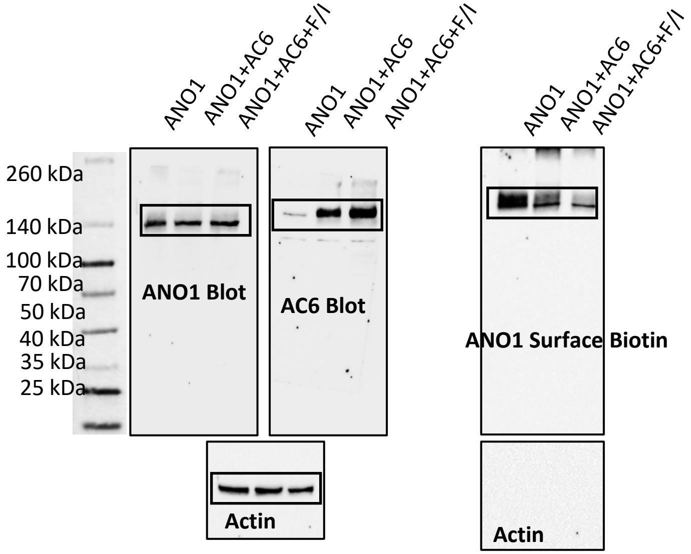

Supplement: Supplementary file 1 — Supplementary Information [file 41467_2025_58682_MOESM1_ESM.pdf]
