# Supplementary material for: Multiple cAMP/PKA complexes at the STIM1 ER/PM junction specified by E-Syt1 and E-Syt2 reciprocally gates ANO1 (TMEM16A) via Ca2+
Source: Nat Commun. 2025 Apr 9;16:3378. doi: 10.1038/s41467-025-58682-w (PMC11982563; doi:10.1038/s41467-025-58682-w)
Supplement: Supplementary file 2 — Reporting Summary [file 41467_2025_58682_MOESM2_ESM.pdf]

nature portfolio

Corresponding author(s): Shmuel Muallem

Last updated by author(s): Feb 13, 2025

Reporting Summary

Nature Portfolio wishes to improve the reproducibility of the work that we publish. This form provides structure for consistency and transparency in reporting. For further information on Nature Portfolio policies, see our Editorial Policies and the Editorial Policy Checklist.

Statistics

For all statistical analyses, confirm that the following items are present in the figure legend, table legend, main text, or Methods section.

n/a	Confirmed	
☐	☒	The exact sample size (*n*) for each experimental group/condition, given as a discrete number and unit of measurement
☐	☒	A statement on whether measurements were taken from distinct samples or whether the same sample was measured repeatedly
☐	☒	The statistical test(s) used AND whether they are one- or two-sided<br>*Only common tests should be described solely by name; describe more complex techniques in the Methods section.*
☒	☐	A description of all covariates tested
☒	☐	A description of any assumptions or corrections, such as tests of normality and adjustment for multiple comparisons
☐	☒	A full description of the statistical parameters including central tendency (e.g. means) or other basic estimates (e.g. regression coefficient) AND variation (e.g. standard deviation) or associated estimates of uncertainty (e.g. confidence intervals)
☒	☐	For null hypothesis testing, the test statistic (e.g. *F*, *t*, *r*) with confidence intervals, effect sizes, degrees of freedom and *P* value noted<br>*Give P values as exact values whenever suitable.*
☒	☐	For Bayesian analysis, information on the choice of priors and Markov chain Monte Carlo settings
☒	☐	For hierarchical and complex designs, identification of the appropriate level for tests and full reporting of outcomes
☒	☐	Estimates of effect sizes (e.g. Cohen's *d*, Pearson's *r*), indicating how they were calculated

Our web collection on statistics for biologists contains articles on many of the points above.

Software and code

Policy information about availability of computer code

Data collection	pClamp 11 and Origin software for current recording; Nikon FluoView software (FV10-ASW 4.2) for confocal images capture and FRET measurements
Data analysis	pClamp 11 and Origin software were used for current analysis; Prism for data and statistical analysis; ImageJ for TIRF puncta analysis and western blot bend intensity determination; Nikon FluoView software (FV10-ASW 4.2) for confocal images analysis a; GraphPad 10 for FRET image analysis and presentation.

For manuscripts utilizing custom algorithms or software that are central to the research but not yet described in published literature, software must be made available to editors and reviewers. We strongly encourage code deposition in a community repository (e.g. GitHub). See the Nature Portfolio guidelines for submitting code & software for further information.

Data

Policy information about availability of data

All manuscripts must include a data availability statement. This statement should provide the following information, where applicable:

- Accession codes, unique identifiers, or web links for publicly available datasets
- A description of any restrictions on data availability
- For clinical datasets or third party data, please ensure that the statement adheres to our policy

All data is available upon request as Prism and/or Excel files.

Research involving human participants, their data, or biological material

Policy information about studies with human participants or human data. See also policy information about sex, gender (identity/presentation), and sexual orientation and race, ethnicity and racism.

Reporting on sex and gender	N/A
Reporting on race, ethnicity, or other socially relevant groupings	N/A
Population characteristics	N/A
Recruitment	N/A
Ethics oversight	N/A

Note that full information on the approval of the study protocol must also be provided in the manuscript.

Field-specific reporting

Please select the one below that is the best fit for your research. If you are not sure, read the appropriate sections before making your selection.

☒ Life sciences ☐ Behavioural & social sciences ☐ Ecological, evolutionary & environmental sciences

For a reference copy of the document with all sections, see nature.com/documents/nr-reporting-summary-flat.pdf

Life sciences study design

All studies must disclose on these points even when the disclosure is negative.

Sample size	All experiments used at least 3 biological replicates and collection of multiple samples from each biological replicate and until statistical significance can be ascertain.
Data exclusions	No data of full experimental protocols were excluded.
Replication	All experiments used at least 3 biological replicates and collection of multiple samples from each biological replicate and until statistical significance can be ascertain. There were no experiments that could not be reproduced.
Randomization	N/A for experiments with transfected cell with tags used to identify the cells.
Blinding	N/A

Reporting for specific materials, systems and methods

We require information from authors about some types of materials, experimental systems and methods used in many studies. Here, indicate whether each material, system or method listed is relevant to your study. If you are not sure if a list item applies to your research, read the appropriate section before selecting a response.

Materials & experimental systems

n/a	Involved in the study	
☐	☒	Antibodies
☐	☒	Eukaryotic cell lines
☒	☐	Palaeontology and archaeology
☐	☒	Animals and other organisms
☒	☐	Clinical data
☒	☐	Dual use research of concern
☒	☐	Plants

Methods

n/a	Involved in the study	
☒	☐	ChIP-seq
☒	☐	Flow cytometry
☒	☐	MRI-based neuroimaging

Antibodies

Antibodies used	Anti-GFP antibodies (#2273763, Invitrogen), anti-myc antibodies (#2276, Cell Signaling technology), anti-flag antibodies (#B3111, Millipore Sigma), anti-HA antibodies (#2367, Cell Signaling technology).

Validation	Specificity of Antibodies were verified by vendor a using plasmids coding for the proteins in transfected cells.

Eukaryotic cell lines

Policy information about cell lines and Sex and Gender in Research

Cell line source(s)	HEK293T Cells were from (ATCC® CRL-3216); NS-SV-AC
Authentication	HEK by ATCC; NS_SA-AC by the provided Dr. John Chiorini NIDCR
Mycoplasma contamination	All cell lines were routinely verified to be free of mycoplasma contamination.
Commonly misidentified lines (See ICLAC register)	N/A

Animals and other research organisms

Policy information about studies involving animals; ARRIVE guidelines recommended for reporting animal research, and Sex and Gender in Research

Laboratory animals	8-week-old C57 mice
Wild animals	N/A
Reporting on sex	Female and male mice
Field-collected samples	N/A
Ethics oversight	The animal use committee of the National Institute of Dental and Craniofacial Research, protocol # 19-882

Note that full information on the approval of the study protocol must also be provided in the manuscript.
